# DNase 2 Is the Main DNA-Degrading Enzyme of the Stratum Corneum

**DOI:** 10.1371/journal.pone.0017581

**Published:** 2011-03-01

**Authors:** Heinz Fischer, Jennifer Scherz, Sandra Szabo, Michael Mildner, Charaf Benarafa, Alicia Torriglia, Erwin Tschachler, Leopold Eckhart

**Affiliations:** 1 Department of Dermatology, Medical University of Vienna, Vienna, Austria; 2 Theodor Kocher Institute, University of Bern, Bern, Switzerland; 3 INSERM UMR 872, Physiopathologie des maladies oculaires, Centre de Recherches des Cordeliers, Paris, France; 4 Centre de Recherches et d'Investigations Épidermiques et Sensorielles, Neuilly-sur-Seine, France; University of Illinois at Chicago, United States of America

## Abstract

The cornified layer, the stratum corneum, of the epidermis is an efficient barrier to the passage of genetic material, i.e. nucleic acids. It contains enzymes that degrade RNA and DNA which originate from either the living part of the epidermis or from infectious agents of the environment. However, the molecular identities of these nucleases are only incompletely known at present. Here we performed biochemical and genetic experiments to determine the main DNase activity of the stratum corneum. DNA degradation assays and zymographic analyses identified the acid endonucleases L-DNase II, which is derived from serpinB1, and DNase 2 as candidate DNases of the cornified layer of the epidermis. siRNA-mediated knockdown of serpinB1 in human *in vitro* skin models and the investigation of mice deficient in serpinB1a demonstrated that serpinB1-derived L-DNase II is dispensable for epidermal DNase activity. By contrast, knockdown of DNase 2, also known as DNase 2a, reduced DNase activity in human *in vitro* skin models. Moreover, the genetic ablation of DNase 2a in the mouse was associated with the lack of acid DNase activity in the stratum corneum *in vivo*. The degradation of endogenous DNA in the course of cornification of keratinocytes was not impaired by the absence of DNase 2. Taken together, these data identify DNase 2 as the predominant DNase on the mammalian skin surface and indicate that its activity is primarily targeted to exogenous DNA.

## Introduction

The outermost layer of the epidermis, i.e. the stratum corneum, is an important barrier of the body to the environment [1], [2]. Both the entry of substances from the environment into the skin and the release of substances from the skin is tightly controled. The stratum corneum consists of dead corneocytes that are devoid of genetic material, however multiple active enzymes are present inside corneocytes and in the intercorneocyte space [1], [3], [4]. DNase activities on the skin surface have already been described in the early 1960s [5], [6]. It has been proposed that the DNases of the stratum corneum have a primary function in the degradation of endogenous DNA during cornification of keratinocytes [6]. In addition, these DNases may play a role in the defense against infectious agents containing genetic information encoded by DNA such as papilloma viruses or bacteria that utilize extracellular DNA for biofilm formation [7]. Recently the interest in the metabolism of DNA in the epidermis has been raised by the finding that extracellular DNA, in conjunction with cathelicidin, is able to activate dendritic cells, which could contribute to the etiology of psoriasis [8]. In spite of the long history of research on epidermal DNases, the molecular identities of DNases in the epidermis and specifically in the stratum corneum have remained largely unknown.

DNases have been classified into DNase I enzymes, which have an activity optimum at approximately pH 7.0 and require magnesium ions, and DNase II enzymes, which have a magnesium-independent activity optimum at approximately pH 5.0. Molecular studies have identified many DNases that are active at neutral pH. Among them are caspase-activated DNase (CAD) [9], endonuclease G [10], [11] and members of the DNase 1 family. The latter comprise DNase 1, DNase 1-like 1 (DNase1L1), DNase1L2 and DNase1L3 [12]. DNase 1 and DNase1L3 are expressed in a wide variety of tissues whereas expression of DNase1L1 is essentially restricted to muscles [13] and DNase1L2 is confined to keratinocytes [14]. DNase1L2 is present in the stratum corneum and, unlike other members of the DNase 1 family, has an activity optimum at pH 5.6 [15], [12]. Furthermore, exonucleases such as TREX1 [16] and TREX2 [17] degrade DNA at neutral pH.

Three acid DNases have been identified in mammals so far. DNase 2a, usually referred to as DNase 2, is a ubiquitous lysosomal enzyme which degrades DNA of phagocytosed apoptotic bodies or DNA entering the cell via endocytosis [18], [19]. DNase 2b, also known as DNase 2-like acid DNase, DLAD, is specifically expressed in the eye and degrades nuclear DNA in the course of terminal differentiation of lens fiber cells [20]. Leukocyte elastase inhibitor (LEI)-derived DNase II or L-DNase II, is generated upon proteolytic processing of the serine protease inhibitor serpinB1, also known as monocyte/neutrophil elastase inhibitor (MNEI) or LEI [21], [22], which is expressed in many cell types including keratinocytes [23]. L-DNase II has been implicated in apoptosis [24]–[26]. Since the pH of mammalian stratum corneum is within the range from pH 4.5 to pH 6 [27], [28], acid DNases are more likely than neutral DNases to be active within the stratum corneum.

In this study we identify the main DNase of the mammalian skin surface and demonstrate that the function of this DNases is not related to the degradation of nuclear DNA during cornification. Our findings may have implications for the defense function of the stratum corneum and for topical gene delivery into the skin.

## Results

### The main DNase of human stratum corneum has DNase II-like properties

**Figure 1:**
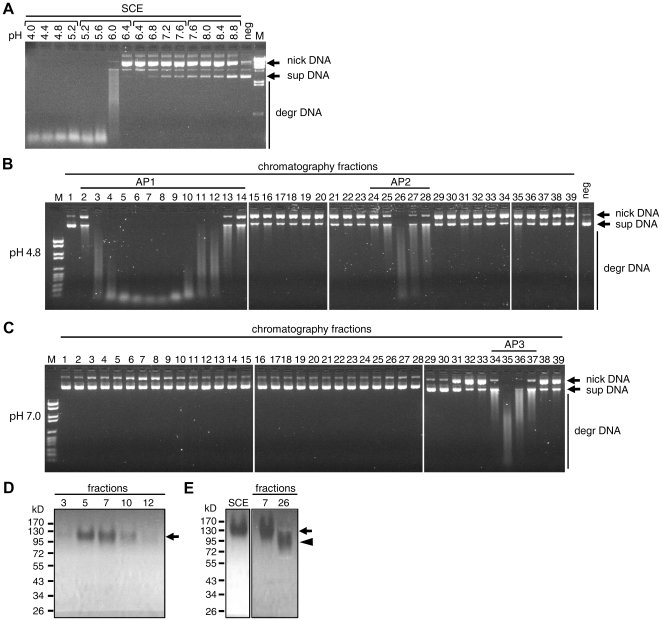
The main DNase of human stratum corneum has DNase II-like properties. (**A**) pH dependence of DNase activity in a stratum corneum extract (SCE). Horizontal brackets indicate incubations that used the same buffer system to adjust different pH values. (**B, C**) An extract from human stratum corneum was fractionated by anion exchange FPLC and the DNase activities of the individual fractions were determined at pH 4.8 (**B**) and at pH 7.0 (**C**). Plasmid DNA was used a substrate for the DNase assays shown in panels **A**–**C**. (**D**) Zymography of fractions corresponding to activity peak AP1. The samples were electrophoresed through a polyacrylamide gel containing DNA. The gel was incubated with ethidium bromide to visualize DNA (white background). DNase activity is indicated by the disappearance of DNA in the gel (black). The molecular weight of the marker proteins is shown on the left side. kD, kilodalton. (**E**) Zymography of total stratum corneum extract and chromatography fractions 7 and 26 which correspond to activity peaks AP1 and AP2, respectively. An arrow and an arrowhead indicate positions of active DNases in the gel. nick DNA, nicked DNA; sup DNA, supercoiled DNA; degr DNA, degraded DNA.

Since conflicting data have been published about the relative contribution of acid and neutral DNases to the total DNase activity on the skin surface [5], [6], we tested the DNase activity of human stratum corneum extracts in the pH range from pH 4 to pH 9. Although the experiment was performed in the presence of magnesium ions to permit the activity of DNase I-type enzymes, the DNase activity was virtually restricted to pH values smaller than pH 6 (Figure 1A). The same result was obtained for stratum corneum from the forearm (Figure 1A) and from the heel (Supplementary Figure S1A).

To determine the minimum number of DNases present in the stratum corneum, we separated a protein extract from human stratum corneum by anion exchange chromatography and tested DNase activities of the fractions. Three DNase activity peaks, termed here AP1 through AP3, were detected (Figure 1B, C). AP1 passed the anion exchange column in the flow-through, but bound to the resin of a cation exchange column (not shown), whereas AP2 and AP3 were retained in the anion exchange column.

AP1 and AP2 were active at acidic pH (Figure 1B) whereas AP3 was active at neutral pH (Figure 1C). AP1 contained by far the most activity. The activity of AP3 could be blocked by omission of magnesium and calcium ions or by the addition of zinc ions (Supplementary Figure S1B), indicating that this activity was mediated by a DNase I-type enzyme. By contrast, AP1 and AP2 showed more activity in the presence of divalent ions but also cleaved DNA in the absence of these ions. This pattern was compatible with DNase II-type identities of AP1 and AP2.

The peak activity fractions were analyzed by gel zymography. In this assay, the proteins were separated under semi-denaturing conditions and DNase activity was visualized by ethidium bromide staining of DNA in the gel, thus marking areas of DNA degradation as dark zones. AP1 contained a single DNase activity band that migrated at an apparent molecular weight of approximately 130 kD (Figure 1D). The strength of this band in the various fractions correlated with the total acid DNase activity of these fractions (Figure 1B), suggesting that AP1 contained only one predominant DNase. AP2 also contained a single DNase band, which consistently migrated slightly faster than the AP1 band (Figure 1E). AP3 did not show activity under the conditions of our zymography assay.

The DNase zymography band corresponding to AP1 was also abundant in unfractionated stratum corneum extract (Figure 1E). Western blot analysis showed that the band did not contain DNase1L2 (Supplementary Figure S1C). In an attempt to identify candidate DNases, we concentrated this band by repeated DNase zymography gel electrophoresis, as described in the Materials and Methods section, and excised the DNase band for subsequent peptide analysis.

### SerpinB1/L-DNase II comigrates with the predominant acid DNase activity of the stratum corneum but is dispensable for its activity

LC-MS/MS analysis of the proteins in the AP1 band revealed the identity of proteins present in this band (Supplementary Table S1). Most peptide hits were derived from bleomycin hydrolase, a cysteine protease that is highly expressed in differentiated keratinocytes but is functionally unrelated to DNases [29]. Among proteins present in the zymography band, we identified serpinB1, the precursor of the acid DNase, L-DNase II (Supplementary Table S1 and Supplementary Figure S2A). No other candidate protein with reported DNase activity was identified among the hits from the stratum corneum extract.

**Figure 2:**
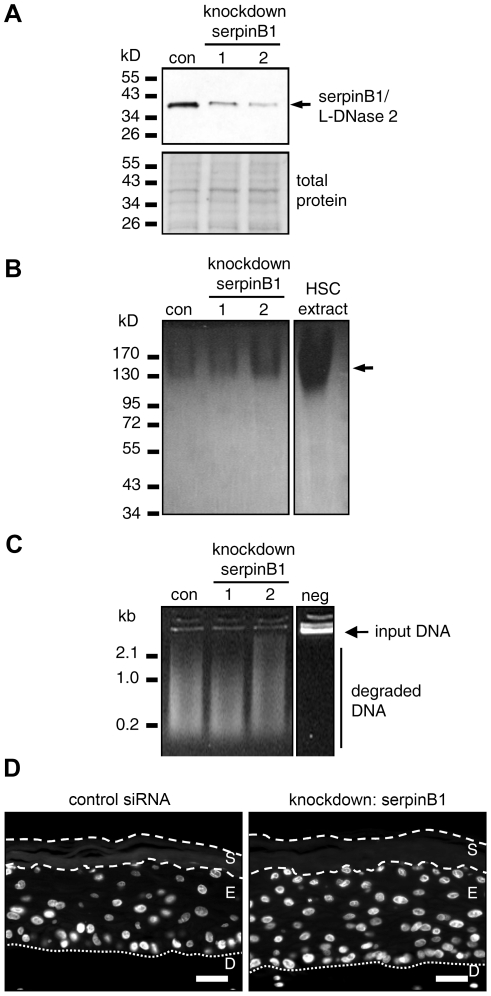
SerpinB1, the precursor of L-DNase II, is not required for acidic DNase activity in human skin models. (**A**) Western blot analysis of serpinB1in skin equivalents. The expression of serpinB1/L-DNase II was knocked down by specific short interfering RNAs (siRNA1, siRNA2) in human skin models *in vitro*. The skin equivalents were lysed and subjected to Western blot analysis using an antibody specific for serpinB1 (top panel). Equal loading was confirmed by Ponceau staining of the same membrane (bottom panel). con, control siRNA. (**B**) DNase zymography of lysates from skin equivalents. (**C**) DNase activity of lysates from skin equivalents was determined at pH 4.8. Lysates were incubated with Lambda DNA. DNA degradation was visualized by gel electrophoresis and ethidium bromide staining. neg, Lambda DNA incubated with lysis buffer. kb, kilo-basepairs. (**D**) Labeling of nuclear DNA with Hoechst 33258 in thin sections of skin equivalents. Fluorescent labeling of DNA is shown in white. The borders of the stratum corneum are marked by discontinuous lines and the border of the epidermis and dermis is marked by a dotted line. S, stratum corneum; E, epidermis; D, dermis; Scale bars, 40 µm.

To test whether serpinB1/L-DNase II contributes to the acid DNase activity of epidermal keratinocytes, we knocked down the expression of serpinB1 with specific siRNAs in human organotypic skin cultures and compared the stratum corneum formation and DNase activities of these cultures with that of cultures treated with control siRNA. Western blot analysis showed that the siRNAs reduced the expression of serpinB1 by approximately 75% (Figure 2A). In zymography, the main DNase activity of *in vitro* skin models was detected at the same position as that in the stratum corneum of human skin (Figure 2B). However, suppression of serpinB1 did not reduce the acid DNase activity of the *in vitro* skin cultures (Figure 2B, C). Histological analysis by hematoxylin and eosin (H&E) staining (Supplementary Figure S2B) and fluorescent labeling of DNA by Hoechst 33258 (Figure 2D) demonstrated that skin models, in which the expression of serpinB1 was suppressed, were able to completely degrade nuclear DNA during stratum corneum formation. These data did not support the hypothesis of a contribution of serpinB1/L-DNase II to the DNase activity of the *in vitro* model of human epidermis. However, since a complete knockdown of serpinB1could not be achieved by siRNA, this experiment did not exclude a role of this enzyme in epidermal DNA degradation.

**Figure 3:**
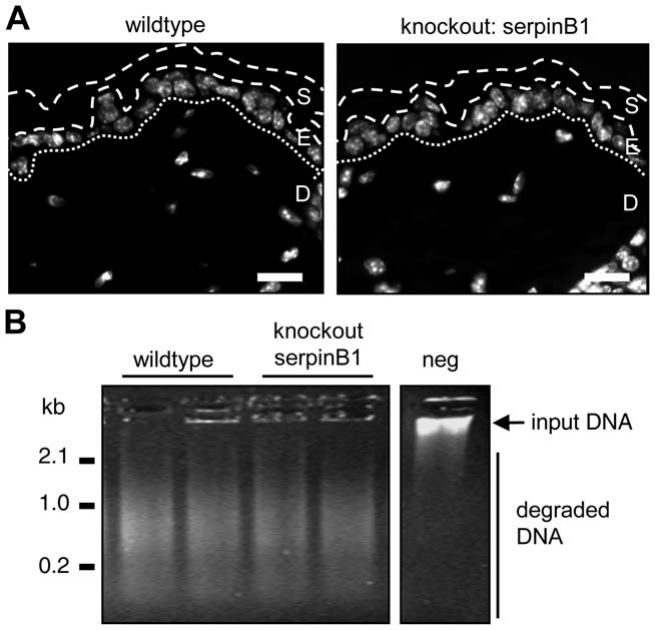
SerpinB1a is not required for acidic DNase activity in murine epidermis. (**A**) Hoechst labeling of DNA in the interfollicular epidermis of wild-type and serpinB1a knockout mice. The borders of the stratum corneum and the border of the epidermis and dermis are marked with discontinuous and dotted lines, respectively. S, stratum corneum; E, epidermis; D, dermis; scale bars, 20 µm. (**B**) Acid DNase activity in extracts from the stratum corneum of the soles of a wild-type and a serpinB1a knockout mouse. 2 extracts were prepared from each mouse and incubated with Lambda DNA that was subsequently electrophoresed in the presence of ethidium bromide. The results are representative of investigations of 3 mice per genotype. kb, kilo-basepairs.

To test the potential role of serpinB1/L-DNase II *in vivo*, we investigated mice deficient in serpinB1a, the functional homologue of human serpinB1 in the murine epidermis (Supplementary Figure S2C). SerpinB1a-deficient mice are morphologically largely normal but were shown to have increased susceptibility to pulmonary infection by *Pseudomonas aeruginosa* [30]. However, the phenotype in tissues other than the lung has not been reported at the microscopic or biochemical level. H&E staining (Supplementary Figure S2D) and *in situ* fluorescent labeling of DNA by Hoechst 33258 (Figure 3A) showed that the stratum corneum of both types of mice was orthokeratotic. Furthermore, stratum corneum preparations from the soles of both genotypes of mice had the same DNase activity at acidic pH (Figure 3B). Similarly, there was no difference in acidic DNase activity in eluates from the surface of hair, which contains both squames from the stratum corneum and sebum (not shown). These findings strongly suggested that L-DNase II does not play a significant role in murine stratum corneum.

### DNase 2 is the main acid DNase of human skin equivalents and essential for acid DNase activity in the stratum corneum of the mouse

Our DNase activity profiling experiments (Fig. 1) suggested that the main DNase of the stratum corneum was a DNase II-type enzyme with an acidic pH optimum. Of the three main acid DNases [25], i.e. serpinB1-derived L-DNase II, DNase 2a, and DNase 2b, L-DNase II was shown to have no role in epidermal DNA degradation by the experiments described above and DNase 2b is not expressed in the epidermis [14]. Together, these data suggested DNase2a, generally referred to as DNase 2, as the major candidate for a role as DNA-degrading enzyme in the stratum corneum.

**Figure 4:**
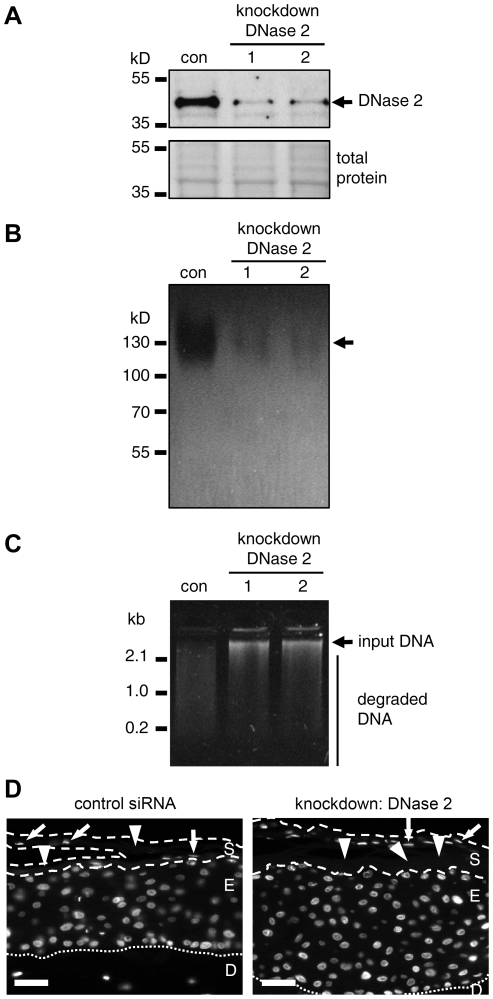
siRNA-mediated knockdown of DNase 2 reduces the main acid DNase activity of a human skin equivalent. The expression of DNase 2 was knocked down by specific short interfering RNAs (siRNA1, siRNA2) in human skin models *in vitro*. (**A**) The skin equivalents were lysed and subjected to Western blot analysis using an antibody specific for DNase 2 (top panel). Equal loading was confirmed by Ponceau staining of the same membrane (bottom panel). con, control siRNA. (**B**) DNase zymography of lysates from skin equivalents. (**C**) DNase activity of lysates from skin equivalents was determined at pH 4.8. Lysates were incubated with Lambda DNA. DNA degradation was visualized by gel electrophoresis and ethidium bromide staining. kb, kilo-basepairs. (**D**) Labeling of nuclear DNA with Hoechst 33258 in thin sections of skin equivalents. Fluorescent labeling of DNA is shown in white. The borders of the stratum corneum are marked by discontinuous lines and the border of the epidermis and dermis is marked by dotted line. Arrows point to nuclear remnants occurring sporadically in the stratum corneum. Arrowheads point to stratum corneum free of nuclear remnants. S, stratum corneum; E, epidermis; D, dermis; Scale bars, 40 µm.

Human epidermal keratinocytes were cultured in 3-dimensional *in vitro* skin models. Zymography showed that these skin models contained a DNase with the same properties as those found in the stratum corneum of human skin. When the expression of the *DNASE2* gene was suppressed by siRNA-mediated knockdown, the predominant acid DNase band was clearly diminished as compared to skin models treated with control siRNA, suggesting that DNase 2 was essential for this activity (Figure 4A, B). Moreover, the total acid DNase activity of lysates from the skin models was reduced by the knockdown of DNase 2 (Figure 4C).

H&E staining suggested that the knockdown of DNase 2 did not impair terminal differentiation of keratinocytes (Supplementary Figure S3A). *In situ* labeling of DNA with Hoechst dye revealed parakeratotic patches in the stratum corneum of the *in vitro* skin models. However, the stratum corneum was largely orthokeratotic with and without knockdown of DNase 2 (Figure 4D, arrowheads). This indicated that DNase 2 was not essential for the degradation of endogenous nuclear DNA during cornification of human keratinocytes.

**Figure 5:**
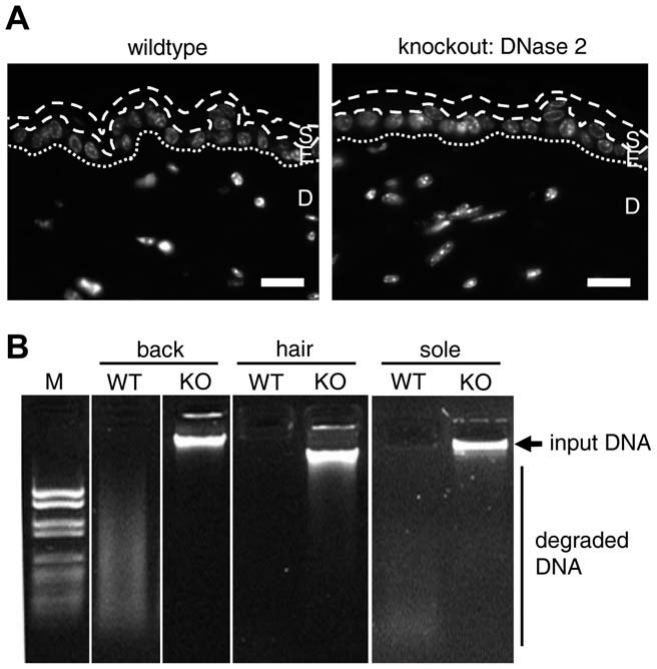
Acid DNase activity is strongly reduced in the stratum corneum of DNase 2-deficient mice. (**A**) Hoechst labeling of DNA in the interfollicular epidermis of mice expressing DNase 2a (wild-type, WT) and of DNase 2a-deficient (knockout, KO) mice. The borders of the stratum corneum and the border of the epidermis and dermis are marked by discontinuous and dotted lines, respectively. S, stratum corneum; E, epidermis; D, dermis; scale bars, 20 µm. (**B**) Acid DNase activity in extracts from the stratum corneum of the back and the soles as well as from the surface of hair of WT and KO mice. M, molecular weight marker VI (Roche). Note that all samples shown here were derived from mice deficient in the interferon type I receptor.

Next we investigated the contribution of DNase 2 to stratum corneum DNase activity *in vivo*. Homozygous DNase 2a-deficiency is embryonic lethal in the mouse [31], [32]. However, the viability can be rescued by concomitant ablation of the interferon type I receptor (*Ifnar*) gene. Mice deficient in both DNase 2a and IFN-IR are phenotypically normal under specific pathogen-free housing conditions except for the development of rheumatoid arthritis at an age of approximately 3 months [33], [34]. Therefore, we analyzed *Ifnar* ^−/−^ *DNase2a* ^−/−^ mice and *Ifnar*−/− *DNase2a*+/− mice. The epidermis of DNase2a-deficient mice was orthokeratotic (Figure 5A and Supplementary Figure S3B), i.e. it did not contain nuclear remnants. These data suggested that DNase2a was dispensable for the breakdown of DNA during cornification of keratinocytes *in vivo*.

To determine the contribution of DNase 2 to the DNase activity of the skin surface, extracts from the stratum corneum of the soles and from the surface of hair of DNase 2a-deficient and control mice were analyzed in an *in vitro* DNA degradation assay. Similar to samples from the human skin surface, stratum corneum and hair extracts of mice expressing wild-type DNase 2a degraded DNA completely (Figure 5B). By contrast, acid DNase activity was absent from the stratum corneum and the surface of hairs of DNase2a-deficient mice (Figure 5B). Taken together, these results demonstrate an essential role of DNase 2a for the acid DNase activity of the stratum corneum and the surface of hair.

## Discussion

This study shows that the stratum corneum contains, besides other minor DNase activities, one predominant DNase activity. Rigorous genetic testing of two candidate acid DNases, i.e. DNase 2 and L-DNase II, demonstrated that DNase 2 plays a non-redundant role as the main DNase of the mammalian stratum corneum whereas L-DNase II is dispensable for the acid DNase activity in the stratum corneum.

Our finding that human stratum corneum extracts contain much stronger DNase activity at acidic pH than at neutral pH is in agreement with previous reports on epidermal DNases [6], [35]. Importantly, the stratum corneum-derived DNase showed activity *in vitro* within a pH range that is compatible with activity at the pH of the stratum corneum of the epidermis, i.e. between pH 4.5 and pH 6 [27], [28]. It is noteworthy that neutral DNases may contribute more significantly to the total DNase activity in other species such as the guinea pig [5], [36]. Our Western blot analysis of human stratum corneum extracts demonstrated that the main DNase of the human stratum corneum was not identical to DNase1L2, which has an activity optimum at pH 5.6 [12] (Suppl. Fig. S1C). Recently, we have knocked out DNase1L2 in the mouse [37]. Deficiency in DNase1L2 resulted in defective degradation of endogenous DNA in hair, nails, tongue and scales of the murine tail [37] whereas DNase activity in the stratum corneum was not compromised by the lack of DNase1L2 (our unpublished data). By contrast, knockout of DNase 2, as described here, caused a dramatic reduction in DNase activity in the murine stratum corneum.

The presence of DNase 2 in the stratum corneum shows that this enzyme survives the conversion of keratinocytes into corneocytes. In living cells DNase 2 resides in the lysosomes where it degrades DNA taken up by endocytosis or phagocytosis [19], [38]. Importantly, lamellar bodies, i.e. the tubovesicular secretory organelles of differentiated keratinocytes in the stratum granulosoum, are related to lysosomes [39]. Therefore, it appears likely that DNase 2 enters the stratum corneum via lamellar bodies. Our results suggest that DNase 2 is active in the stratum corneum and in the material attached to beard hair. The latter contains desquamated corneocytes but also sebum so that a part of its DNase activity may be derived from sebaceous glands. Importantly, the fact that the DNase is present in the stratum corneum of plantar epidermis, which lacks sebaceous glands, demonstrates that sebocytes are not essential for the formation of stratum corneum DNase activity.

Our data suggest that serpinB1-derived L-DNase II contributes neither directly nor indirectly to the DNase activity of the stratum corneum. L-DNase II was originally identified in a commercial preparation of DNase 2 [21], possibly indicating that it can be easily co-purified with DNase 2 or that it might even physically interact with DNase 2. It is tempting to speculate that serpinB1 bound to DNase 2 in a putative multi-protein complex that migrated at the apparent molecular weight of approximately 130 kD in zymography. However, this potential interaction, if it exists, does not appear to be required for the activity of DNase 2, because knockdown of human serpinB1 and knockout of murine serpinB1a did not reduce the acid DNase activity. It is also noteworthy that zymographic analysis of mouse tissues, irrespectively of the *Dnase2a* and *serpinb1a* genotype, did not show any acid DNase band under the conditions that revealed the predominant acid DNase of human tissues and stratum corneum (not shown). This may indicate that the biochemical properties of DNase 2 and its putative binding partner differ between species.

The identification of the main DNase of the stratum corneum facilitates the specific investigation of DNA breakdown as a component of epidermal homeostasis and barrier function. Several processes may be controlled by DNase 2. By targeting DNA of viruses on their inward passage through the skin during infection or on the outward passage through the skin during release of viral particles, DNase 2 may contribute to the antiviral defene of the skin. In addition, DNase 2 is likely to represent a barrier against gene delivery through the skin surface [19]. Furthermore, epidermal DNase 2 may suppress inflammatory reactions by degrading extracellular DNA which, in conjunction with cathelicidin, has the potential to activate dendritic cells [8]. The DNase 2-deficient mice, that were used in the present study, lacked interferon type I receptor to avoid the embryonic lethal effect of constitutive DNase 2 deficiency [34] and therefore were not suitable to study the effect of undegraded DNA on epidermal interferon signaling. Accordingly, an epidermis-specific knockout of DNase 2 in fully immunocompetent mice should be generated for future investigations of the role of DNase 2 in the epidermis. Finally, it will be interesting to test systematically whether the level of DNase 2 activity in the stratum corneum, possibly determined by genetic [40], [41] or environmental factors, is a risk factor for human skin diseases.

## Materials and Methods

### Mice

*Serpinb1a*-deficient mice were generated previously [30] by homologous recombination in W4/129S6 embryonic stem cells derived from 129S6/SvEv/Tac (129S6) mice (Taconic). Tissue samples were obtained after CO_2_-induced euthanasia from three serpinB1a knockout female mice and age-matched wild-type 129S6 female mice under protocols (license number 81/09) approved by the Cantonal Veterinary Office of Bern, Switzerland.

Two *Ifnar* ^−/−^ *DNase2a* ^−/−^, one *Ifnar* ^−/−^ *DNase2a* ^+/−^ and one *Ifnar* ^−/−^ *DNase2a* ^+/+^ mouse were kindly provided by Shigekazu Nagata and Kohki Kawane (Department of Medical Chemistry, Kyoto University Graduate School of Medicine, Yoshida-Konoe, Kyoto, Japan) [34]. The animals were shipped to Vienna and sacrificed by CO_2_-induced euthanasia. Tissue samples were obtained from dead mice. In accordance with the Austrian Law for Animal Experiments, the Ethics Committee for Animal Studies of the Medical University of Vienna confirmed that a specific approval of this study was not required, because no experiments on live animals were performed.

### Preparation of human and murine stratum corneum extracts

Human stratum corneum squames were derived from the heels of healthy volunteers using a callus rasp. Alternatively, hair and stratum corneum squames were collected from the electric shavers of healthy volunteers. Only samples generated by routine cosmetic procedures were used, and data were analyzed anonymously. The Ethics Committee of the Medical University of Vienna waived the need for ethics approval of this study. Murine plantar stratum corneum was scraped off from the soles of euthanized mice using a scalpel. All stratum corneum samples were stored at −80°C. For extraction of soluble proteins, the samples were incubated at 4°C for 30 minutes under rotation using an extraction buffer containing 0.5× PBS, 0.1% Igepal CA-630 (Sigma-Aldrich, St. Louis, MO) including a protease inhibitor cocktail (P8340, Sigma-Aldrich) at a dilution of 1∶1000. The extract was cleared by repeated centrifugation at 20000 g and measured for protein content using the micro BCA protein assay kit (Thermo Scientific, Rockford, IL).

### Chromatographic fractionation of human stratum corneum extracts

The chromatographic separation of stratum corneum proteins was performed using a HiTrap QFF anion exchange column as described previously [42]. The same fractions as in the previous report were analyzed for their DNase activities.

### Gene knockdown in human skin equivalent cultures

The expression of serpinB1 was knocked down in normal human epidermal keratinocytes which were then allowed to differentiate in an *in vitro* organotypic skin model according to a protocol published previously [43]. Briefly, human primary keratinocytes (Cell Systems) were transfected with one of the following double-stranded stealth siRNAs (Invitrogen) using lipofectamine 2000 (Invitrogen): serpinB1 siRNA1 (HSS103168) GCAGGCAUCGCAACUUUCUGCAUGU, serpinB1 siRNA2 (HSS103170) GGCAUGUCAGGAGCCAGAGAUAUUU, control siRNA GAGUGGGUCUGGGUCUUCCCGUAGA, DNase2 siRNA1 GGUCUACAAGCUGCCAGCUCUUAGA, DNase2 siRNA2 CAAGAACCCUGGAACAGCAGCAUCA, control siRNA GGUCAAACGGUCCGAUCUCUUCAGA. From day 1 to day 7 after transfection keratinocytes were maintained in a 3-dimensional culture to facilitate their terminal differentiation and the establishment of a stratum corneum.

### RT-PCR analysis of murine serpinB1 homologs

RNA was prepared from the skin of mouse ears using TRIzol reagent (Invitrogen, Carlsbad, CA) and reverse transcribed according to a published protocol [44]. Two murine homologs of serpinB1 were amplified with primers that anneal to unique sequences in exons 5 and 6 of the respective gene. SerpinB1a was amplified with the primers 5′-AATCCCAGAACTGTTGTCTGT-3′ and 5′- TTCAGGTCCGAAATGTAACCA-3′ to yield a product of 210 bp from cDNA of spliced mRNA and a product of 404 bp from genomic DNA, respectively. SerpinB1b was amplified with the primers 5′-AATCCCAGAACTGCTGGCTAA-3′ and 5′- TTCAGGTCCGAAATGTAACCG-3′ to yield a product of 210 bp from cDNA of spliced mRNA and a product of 401 bp from genomic DNA, respectively.

### Western blot analysis

Western blot analysis was performed according to a previously published protocol [45]. Rabbit anti-serpinB1 [46], a generous gift of Eileen Remold-O'Donnell, was used at a dilution of 1∶4000. Rabbit anti-DNase2 (ProSci) was used at a dilution of 1∶1000. For both primary antibodies, goat anti-rabbit immunoglobulin G conjugated with horseradish peroxidase (Pierce; Product Code: 31463); 1∶10000 in blocking buffer) was used as the second step antibody, and ChemiGlow reagent (Alpha Innotech) was used as a substrate.

### DNA labeling *in situ* and histological analysis

Specimens from human *in vitro* skin models and from murine skin as well as murine nail units were fixed in phosphate-buffered 4.5% formaldehyde and embedded in paraffin. The samples were thin-sectioned and either labeled with Hoechst 33258 (Invitrogen) or stained with H&E as described previously [47].

### Zymography

DNase zymography was performed according to a published protocol [48] with modifications. Briefly, proteins were separated using an SDS-polyacrylamide (4%) stacking gel and a SDS-polyacrylamide (8%) separation gel which was co-polymerized with 20 µg/ml sonicated salmon sperm DNA (Agilent, Santa Clara, CA). For sample loading, the extracted proteins were mixed with an equal volume of 2× non-denaturing loading buffer containing 50 mM Tris-HCl pH 6.8 (Sigma-Aldrich), 2 mg bromophenol blue (Sigma-Aldrich) and 20% glycerol. After the separation, the proteins were re-naturated in 50 mM Tris-HCl pH 7.5, 5 mM MgCl_2_, and 20% 2-propanol for 1 hour at room temperature involving three changes of the buffer. For the DNase reaction, the gel was incubated over night at 37°C with gentle rocking in a buffer containing 3 mM MgCl_2_, 3 mM CaCl_2_, 1 mM 2-mercaptoethanol (Merck, Hohenbrunn, Germany), 50 mM acetic acid-NaOH (pH 4.8), and 0.04% ethidium bromide. Photographs were taken under UV-light after incubation for 30 minutes, 2 and 18 hours.

For preparative zymography, 1 g of hair and stratum corneum squames was collected with an electric shaver and incubated in 25 ml extraction buffer containing 25 mM Tris-HCl, pH 7.4, and protease inhibitors (Complete Mini EDTA free, Roche Applied Science, Mannheim, Germany) under vigorous shaking at 4°C for 1 h. Insoluble material was removed by 2 centrifugation steps at 2000 g for 15 minutes each. The supernatant was sterile-filtered through a 0.2 µm nylon membrane. Twenty ml of this solution were concentrated to 250 µl by subsequent centrifugations in spin-columns with molecular weight limits of 50 kD (Microcon YM-50, Millipore, Bedford, MA) and 3 kD (Centriprep YM-3, Millipore). The protein concentration was measured using the micro-BCA protein assay kit (Thermo Scientific, Rockford, IL). Thirty µg (4 µl) were loaded onto each of the 8 lanes of a zymography gel. After running the gel, a strip of 7 mm width around the apparent molecular weight of 130 kD was excised and proteins were extracted using the EzWay PAG Protein Elution Kit (Komabiotech, Seoul, Korea). Eluted proteins were concentrated to 30 µl using a Microcon YM-30 spin column (Millipore). Twenty-five µl of purified and concentrated proteins were used for zymography and for silver staining. Silver-stained gel bands corresponding to the main DNase band were excised and stored in ultrapure water until LC-MS/MS analysis.

### Liquid chromatography tandem mass spectrometry (LC-MS/MS)

Sample preparation and LC-MS/MS were performed by NextGen Sciences, Inc. (Ann Arbor, MI). Briefly, samples were subjected to proteolytic digestion with trypsin and analyzed using LC-MS/MS with a 30 minute-gradient on a LTQ Orbitrap XL (Thermo Scientific) mass spectrometer. Production data were searched against the concatenated forward and reverse IPI Human database using the Mascot search engine according to the standard protocol of NextGen Sciences.

### DNA digestion assay

DNase activity *in vitro* was measured in stratum corneum extracts (see above) as well as in lysates obtained from skin equivalents. Skin equivalents were grinded with a mortar in liquid nitrogen and lyzed in 0.5× PBS containing 1% Igepal CA-630 (Sigma-Aldrich) and a protease inhibitor cocktail (P8340, Sigma-Aldrich) at a dilution of 1∶500. Insoluble debris was removed by centrifugation in a table centrifuge at 20000 g at 4°C for 20 minutes. The supernatant was stored at −80°C. Two µl of the sample were added to a reaction mix with a total volume of 20 µl containing 0.75 µg Lambda DNA (Roche Applied Science), 3 mM CaCl_2_, 3 mM MgCl_2_, 1 mM 2-mercapto-ethanol, and 50 mM acetate-NaOH (pH 4.8). The mix was incubated at 37°C for 1 or 4 hours and then separated by electrophoresis on a 1% agarose gel containing ethidium bromide. In some experiments plasmid DNA (pCR2.1-TOPO 2.1, Invitrogen) was used instead of Lambda DNA and the pH value was adjusted with the following buffer systems (all 50 mM): acetate-NaOH (pH 4.0–pH 5.2), MES-NaOH (pH 5.2–pH 6.4), MOPS-NaOH (pH 6.4–pH 7.6) and Tris-HCl (pH 7.6–pH 8.8).

## Supporting Information

Table S1 **Proteins identified in the main DNase zymography band of human stratum corneum extract.**
(XLS)

Figure S1 **Characterization of DNase activities in human stratum corneum.** (**A**) The DNase activity of an extract from the stratum corneum (SCE) of the heel was analyzed at different pH values. (**B**) Ion dependence of DNase activities. An extract from human stratum corneum was subjected to anion exchange chromatography as described in the Materials and Methods section. The fractions 7, 26, and 35, corresponding to those shown in Figure 1B, were incubated with plasmid DNA in the presence and absence of magnesium and calcium ions (Mg/Ca), zinc ions (Zn) or ethylenediaminetetraacetic acid (EDTA), followed by agarose gel electrophoresis and visualization of DNA by ethidium bromide staining. (**C**) A hair and stratum corneum extract (HSCE) was separated on a zymography gel as shown in Figure 1E, and then subjected to Western blot analysis using a goat anti-DNase1L2 antiserum as primary antibody. nick DNA, nicked DNA; sup DNA, supercoiled DNA; degr DNA, degraded DNA; M, marker VI (Roche).
(TIF)

Figure S2 **Testing the role of serpinB1-derived L-DNase II in epidermal DNA degradation.** (**A**) Amino acid sequence of serpinB1 showing the 11 peptides (underlined) identified by LC-MS/MS. A stratum corneum extract was subjected to DNase zymography as shown in Figure 1E. The band corresponding to the main DNase activity was excised, extracted, digested with trypsin and analyzed by LC-MS/MS. SerpinB1 was among the proteins from which the most abundant peptides were identified, as listed in Supplementary Table S1. (**B**) Hematoxylin and eosin (H&E) staining of thin sections of skin models made of keratinocytes treated with control siRNA and serpin B1/L-DNaseII-specific siRNA. The skin models were fixed, embedded in paraffin, thin-sectioned and stained with H&E. Scale bars, 40 µm. (**C**) RT-PCR analysis of serpinB1 homologs in the murine epidermis. Four serpinB1 homologs of the human gene have been identified in the mouse but only two of these genes, *serpinb1a* and *serpinb1b*, produce full-length functional serpins [49]. RNA from murine epidermis was subjected to RT-PCR analysis with primers annealing to exons 5 and 6 of serpinB1a and serpinB1b, respectively. The amplification of fully spliced mature mRNA resulted in a gel electrophoresis band of 210 basepairs (bp), which is indicated by an asterisk. The amplification of genomic DNA (gDNA), which was present at low concentrations in both cDNA preparations cDNA1 and cDNA2, resulted in a band of 404 bp for serpinB1a and 401 bp for serpinB1b, respectively (double asterisk). Therefore, the gDNA served as an internal control, and the ratio of the signal of the cDNA-derived band to the signal of the gDNA-derived band could be used to compare the expression levels of serpinB1a and serpinB1b. The serpinB1a PCR yielded a very strong band of 210 bp (cDNA) and no band of 404 bp (gDNA) whereas the serpinB1b PCR yielded a very weak band of 210 bp (cDNA) and a strong band of 401 bp (gDNA). This implied that the copy number of serpinB1a cDNA molecules was much higher than that of serpinB1b cDNA molecules. The same result was obtained for cDNA preparation cDNA2. Lanes M show the DNA size marker VI (Roche). (**D**) H&E staining of skin from wildtype and serpinB1a-deficient mice. Scale bars, 20 µm.
(TIF)

Figure S3 **Testing the role of DNase 2 in epidermal DNA degradation.** (**A**) The expression of DNase 2 was knocked down by siRNAs in human skin models *in vitro*. An siRNA with scrambled sequence was used in the control experiment. The skin models were fixed, embedded in paraffin, thin-sectioned and stained with H&E. Scale bars, 40 µm. (**B**) H&E staining of the epidermis of DNase2a-deficient and control mice. Scale bars, 20 µm.
(TIF)
