# Supplementary figures and images for: DNase 2 Is the Main DNA-Degrading Enzyme of the Stratum Corneum
Source: PLoS One. 2011 Mar 1;6(3):e17581. doi: 10.1371/journal.pone.0017581 (PMC3046983; doi:10.1371/journal.pone.0017581)

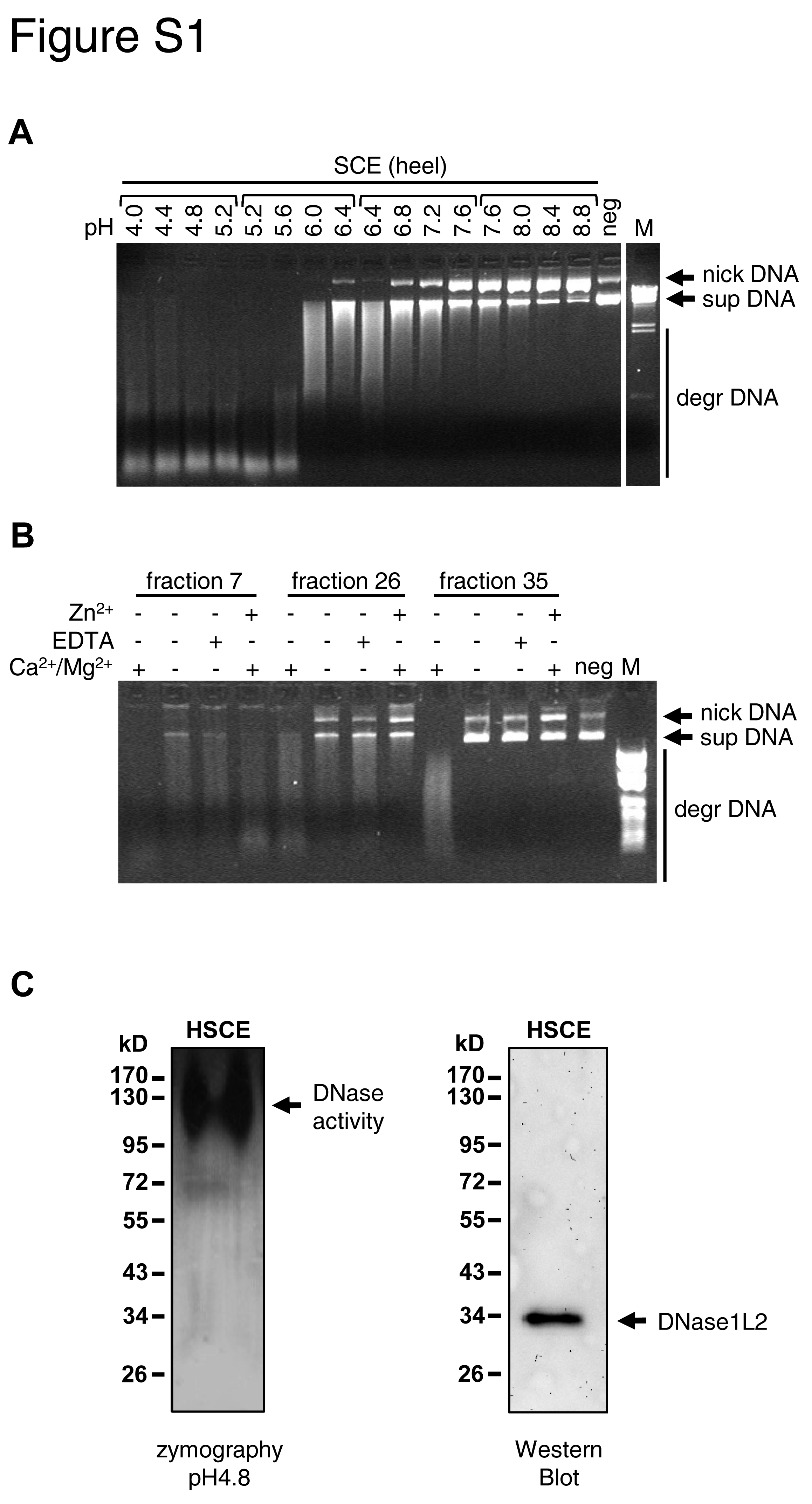

Supplement: Figure S1 — Characterization of DNase activities in human stratum corneum. (A) The DNase activity of an extract from the stratum corneum (SCE) of the heel was analyzed at different pH values. (B) Ion dependence of DNase activities. An extract from human stratum corneum was subjected to anion exchange chromatography as described in the Materials and Methods section. The fractions 7, 26, and 35, corresponding to those shown in Figure 1B, were incubated with plasmid DNA in the presence and absence of magnesium and calcium ions (Mg/Ca), zinc ions (Zn) or ethylenediaminetetraacetic acid (EDTA), followed by agarose gel electrophoresis and visualization of DNA by ethidium bromide staining. (C) A hair and stratum corneum extract (HSCE) was separated on a zymography gel as shown in Figure 1E, and then subjected to Western blot analysis using a goat anti-DNase1L2 antiserum as primary antibody. nick DNA, nicked DNA; sup DNA, supercoiled DNA; degr DNA, degraded DNA; M, marker VI (Roche). (TIF) [file pone.0017581.s002.tif]

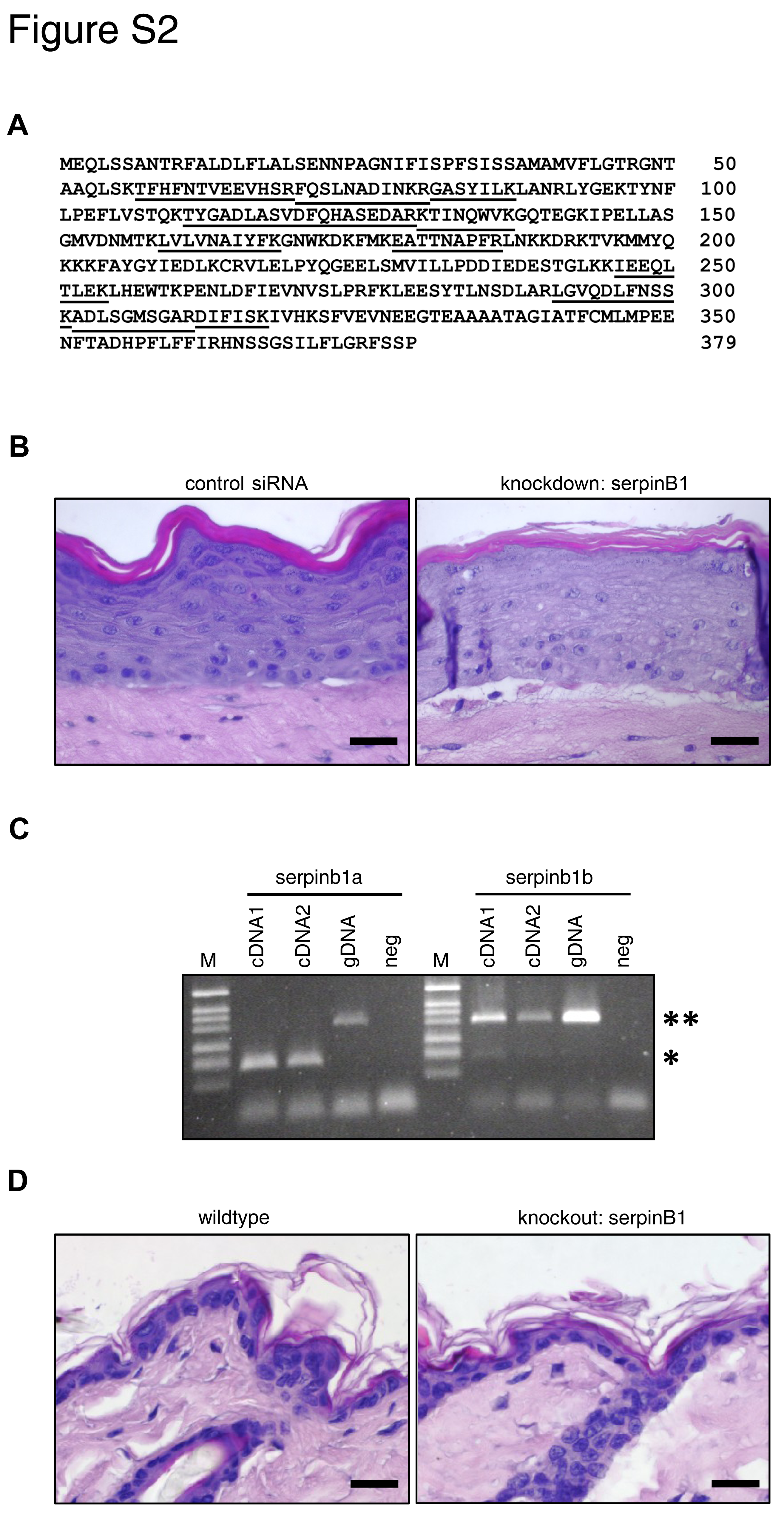

Supplement: Figure S2 — Testing the role of serpinB1-derived L-DNase II in epidermal DNA degradation. (A) Amino acid sequence of serpinB1 showing the 11 peptides (underlined) identified by LC-MS/MS. A stratum corneum extract was subjected to DNase zymography as shown in Figure 1E. The band corresponding to the main DNase activity was excised, extracted, digested with trypsin and analyzed by LC-MS/MS. SerpinB1 was among the proteins from which the most abundant peptides were identified, as listed in Supplementary Table S1. (B) Hematoxylin and eosin (H&E) staining of thin sections of skin models made of keratinocytes treated with control siRNA and serpin B1/L-DNaseII-specific siRNA. The skin models were fixed, embedded in paraffin, thin-sectioned and stained with H&E. Scale bars, 40 µm. (C) RT-PCR analysis of serpinB1 homologs in the murine epidermis. Four serpinB1 homologs of the human gene have been identified in the mouse but only two of these genes, serpinb1a and serpinb1b, produce full-length functional serpins [49]. RNA from murine epidermis was subjected to RT-PCR analysis with primers annealing to exons 5 and 6 of serpinB1a and serpinB1b, respectively. The amplification of fully spliced mature mRNA resulted in a gel electrophoresis band of 210 basepairs (bp), which is indicated by an asterisk. The amplification of genomic DNA (gDNA), which was present at low concentrations in both cDNA preparations cDNA1 and cDNA2, resulted in a band of 404 bp for serpinB1a and 401 bp for serpinB1b, respectively (double asterisk). Therefore, the gDNA served as an internal control, and the ratio of the signal of the cDNA-derived band to the signal of the gDNA-derived band could be used to compare the expression levels of serpinB1a and serpinB1b. The serpinB1a PCR yielded a very strong band of 210 bp (cDNA) and no band of 404 bp (gDNA) whereas the serpinB1b PCR yielded a very weak band of 210 bp (cDNA) and a strong band of 401 bp (gDNA). This implied that the copy number of serpinB1a cDNA molecules was [file pone.0017581.s003.tif]

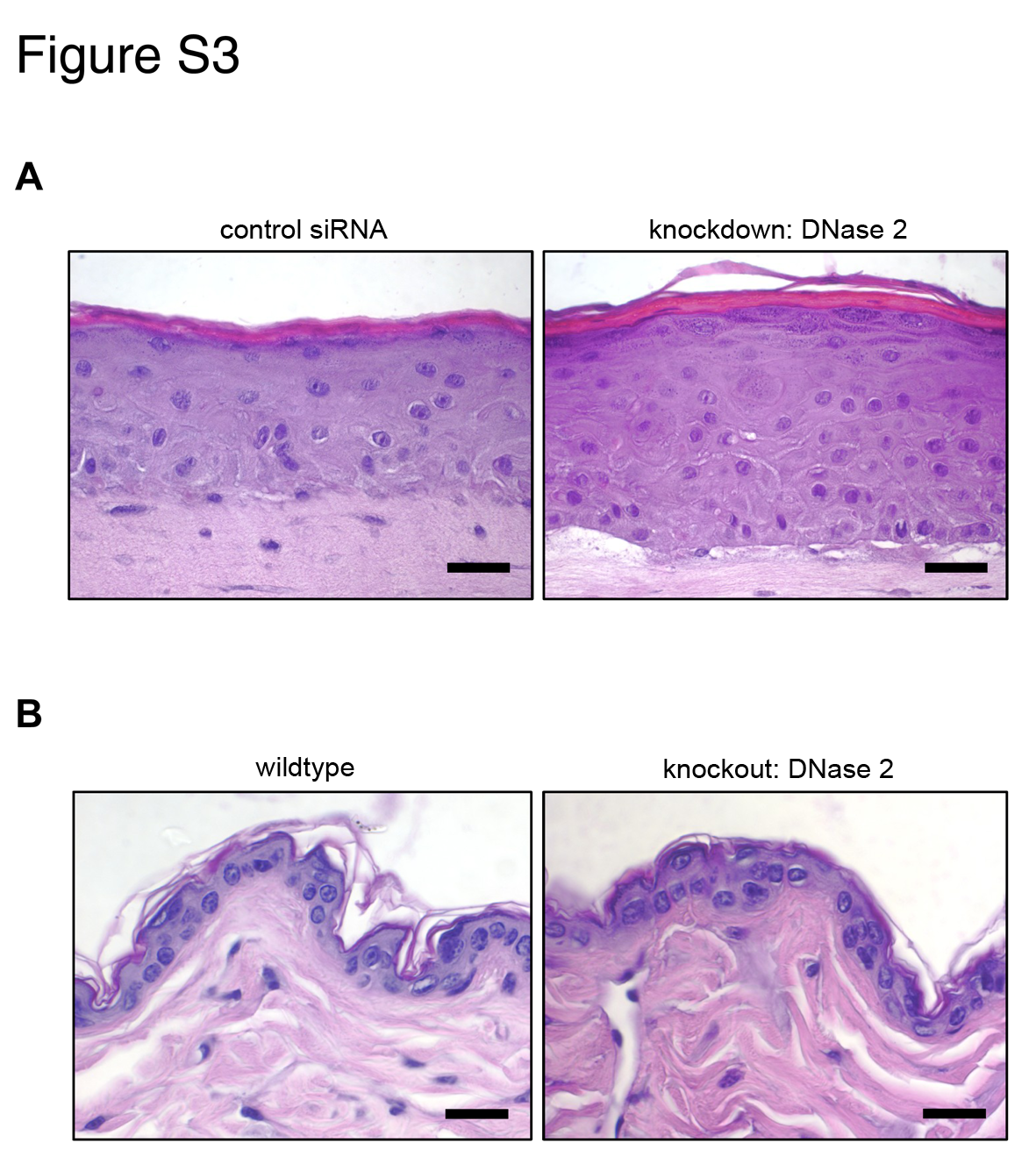

Supplement: Figure S3 — Testing the role of DNase 2 in epidermal DNA degradation. (A) The expression of DNase 2 was knocked down by siRNAs in human skin models in vitro. An siRNA with scrambled sequence was used in the control experiment. The skin models were fixed, embedded in paraffin, thin-sectioned and stained with H&E. Scale bars, 40 µm. (B) H&E staining of the epidermis of DNase2a-deficient and control mice. Scale bars, 20 µm. (TIF) [file pone.0017581.s004.tif]
